# Inflammation in the Disease: Mechanism and Therapies 2014

**DOI:** 10.1155/2015/169852

**Published:** 2015-03-24

**Authors:** M. Seelaender, J. C. Rosa Neto, G. D. Pimentel, R. S. Goldszmid, F. S. Lira

**Affiliations:** ^1^Cancer Metabolism Research Group, Institute of Biomedical Sciences, University of São Paulo (USP), 05508-000 São Paulo, SP, Brazil; ^2^Immunometabolism Research Group, Institute of Biomedical Sciences, University of São Paulo (USP), 05508-000 São Paulo, SP, Brazil; ^3^Department of Internal Medicine, State University of Campinas (UNICAMP), 13083-970 Campinas, SP, Brazil; ^4^Laboratory of Experimental Immunology, Cancer and Inflammation Program, Center for Cancer Research, National Cancer Institute, Bethesda, MD 21702, USA; ^5^Exercise and Immunometabolism Research Group, Department of Physical Education, State University of São Paulo (UNESP), 19060-900 Presidente Prudente, SP, Brazil

Inflammation in the face of harming stimuli protects the organism; as a result, it is an essential route for survival, in which both innate and adaptive immunity are involved. This process must be tightly controlled and terminated in order to warrant the reestablishment of body homeostasis. Therefore, activation of resident inflammatory cells and the recruitment and modulation of migrating inflammatory cells must be ceased. When failure in neutralizing acute inflammation occurs, there is augmented risk of development of chronic inflammation, leading to several metabolic consequences. In the present special issue, original research studies as well as review articles address the inflammatory process as a key contributor to disease onset and progression. In addition, pharmacological and nonpharmacological therapies are examined and the molecular and physiological mechanisms of the treatments are discussed.

Among the 24 accepted papers, 7 approach nutritional therapy and inflammation (J.-Y. Jhun et al.; E. A. Lima et al.; H. B. F. Silva et al.; G. I. G. Souza et al.; B. M. Mohammed et al.; C. A. Morais et al.; S. Arora et al.). The selected papers discuss the effects of Macadamia oil supplementation attenuation of adipocyte hypertrophy and of the inflammatory response of adipose tissue macrophages. The protection induced by supplementation of chitosan coacervate whey protein against metabolic changes and obesity-related inflammation is investigated. Other papers examine Jussara supplementation-associated reversal of the adverse effects of perinatal intake of transfatty acids, the beneficial effect of red genistein extract on autoimmune arthritis, and the role of vitamin C in the resolution of inflammation. Finally, the promotion, by nutritional intervention, of the recovery of glucose homeostasis is discussed. In addition, results on the effect of microencapsulated probiotics on alcoholic liver disease are presented. Taken together, these papers show new encouraging results of nutritional therapy in counteracting the symptoms of obesity and insulin resistance.

The ability of exercise to modulate chronic inflammation was the center of two papers (C. P. Papini et al. and B. Koc et al.,). These studies report that a community-based exercise program results in decrease or maintenance of inflammatory biomarkers after 1 year and thus presents strong potential in public health approaches for chronic disease prevention.

Inflammation and its mechanisms are discussed in the development and progression of many diseases, such as in ectopic fat deposition (L. Liu et al.), acute and chronic kidney disease (P. Ranganathan et al.), and polymicrobial sepsis (G. Pizzino et al.). The role of interleukin- (IL-) 18 in the regulation of toll-like receptors and mannose receptor expression (L. A. Dias-Melicio et al.) is examined. Inflammation in the metabolic syndrome (E. Hopps et al.) and the effect of metformin on autoimmune arthritis (H.-J. Son et al.) are debated. Gut microbiota participation in inflammation is addressed (Mingming Sun et al.; Yu Lijuan et al.), and its regulation by short-chain fatty acids in diabetes (A. Puddu et al.) contemplated. The contribution of circulating LL-37 and inflammatory cytokines in the setting of plaque and guttate psoriasis (Y. J. Hwang et al.) is commented on. Other papers consider ionotropic and metabotropic proton-sensing receptors in allergic asthma (H. Aoki et al.) and autologous bone marrow stem cell transplantation in myocardial infarction (E. B. Furenes et al.). Flavocoxid and infliximab are examined in the context of classical and nutraceutical therapies (A. Bitto et al.; L. Yu et al.). The role of IL-38 and related cytokines in inflammation (X. Yuan et al.) is discussed, in addition to gingival inflammation and pregnancy (M. Wu et al.) and the contribution of the immune system in triplet repeat expansion diseases (M. Olejniczak et al.).

Collectively, this issue provides insight on the role of acute and chronic inflammation in different diseases and discusses mechanisms and new treatment strategies.

*M. Seelaender* *M. Seelaender* *J. C. Rosa Neto* *J. C. Rosa Neto* *G. D. Pimentel* *G. D. Pimentel* *R. S. Goldszmid* *R. S. Goldszmid* *F. S. Lira* *F. S. Lira*

